# Characteristics of the Seed Germination and Seedlings of Six Grape Varieties (*V. vinifera*)

**DOI:** 10.3390/plants11040479

**Published:** 2022-02-10

**Authors:** Zhi-Lei Wang, Miao Hui, Xue-Qing Shi, Dong Wu, Ying Wang, Xing Han, Xiao Cao, Fei Yao, Hua Li, Hua Wang

**Affiliations:** 1 College of Enology, Northwest A&F University, Yangling 712100, China; wangzhilei@nwafu.edu.cn (Z.-L.W.); huimiao688@nwafu.edu.cn (M.H.); xueqingshi@nwafu.edu.cn (X.-Q.S.); wudong@nwafu.edu.cn (D.W.); wangying2018@nwafu.edu.cn (Y.W.); hanxing@nwafu.edu.cn (X.H.); caoxiao@nwafu.edu.cn (X.C.); yaofei@nwafu.edu.cn (F.Y.); 2 China Shaanxi Engineering Research Center for Viti-Viniculture, Yangling 712100, China; 3 China Wine Industry Technology Institute, Yinchuan 750021, China; 4 Engineering Research Center for Viti-Viniculture, National Forestry and Grassland Administration, Yangling 712100, China

**Keywords:** *V. vinifera*, grape seeds, seed germination, seedling formation

## Abstract

Intraspecific recurrent selection in *V. vinifera* is an effective method for breeding of high quality, disease-, cold-, and drought-resistance grapes. Exploring the optimal treatment methods for grape (*V. vinifera*) seeds can help to accelerate the process of intraspecific recurrent selection and improve breeding efficiency. In this study, seeds of six *V. vinifera* varieties were used as experimental materials, and the germination and seedling formation characteristics were studied by single factor treatment and orthogonal compound treatment, respectively. To do this, stratification, chemical substances, beak cutting, and pre-germination treatments were tested, and the optimal treatment combination was determined for each variety. The results indicated that the optimal conditions obtained in the orthogonal experiments were not completely consistent with those in the single-factor experiments. Single factor experiment results demonstrated that two stratification methods (chilling gauze-storage and chilling sand-storage) and two pre-germination methods (pre-germination in petri dishes and pre-germination in a bean sprouter) vary in effectiveness for different varieties. gibberellin acid (GA_3_) soaking and beak-cutting promote the germination and seedling rate of the tested varieties. Orthogonal test results demonstrate that, for Dunkelfelder and Cabernet Sauvignon, the optimal treatment combination was chilling sand-storage + GA_3_ soaking seed + beak cutting + pre-germination in petri dishes. For Meili, the optimal treatment combination was chilling sand-storage + acetic acid (HAc) soaking seed + beak cutting + pre-germination in petri dishes. For Ecolly, the optimal treatment combination was chilling sand-storage + GA_3_ soaking seed + beak cutting + pre-germination in a bean sprouter. For Garanior, the optimal treatment combination was chilling sand-storage + HAc soaking seed + no beak cutting + pre-germination in petri dishes. For Marselan, the optimal treatment combination was chilling gauze-storage + GA_3_ soaking seed + beak cutting + pre-germination in a bean sprouter. This study identified the optimal conditions for seed germination and seedling formation of six grape varieties, which will facilitate future work to characterize the seed germination and seedling formation of seeds obtained by intraspecific hybridization of these varieties. This work also provides a reference for addressing problems of low seed germination rate and suboptimal seedling formation for better utilization of grape germplasms.

## 1. Introduction

Grapes have been cultivated and bred for more than 200 years. The successful breeding of different grape varieties for different purposes requires high-quality grape genetic resources and typically a variety of breeding methods and techniques have been used. Breeding is done to improve the quality of grapes, including enhanced resistance of plants to biological and abiotic stress or an extended or shortened maturity period to meet market needs [1]. Hybrid breeding and seed selection are important aspects of the breeding process [2]. As the most widely cultivated grape with the highest economic value in the world, *V. vinifera* is widely used as the main material for breeding new grape varieties [3,4]. Intraspecific recurrent selection in *V. vinifera* is an effective method for breeding of high quality, disease-, cold-, and drought-resistance grapes [4]. However, more effective breeding strategies require improved germination and seedling formation rates of intraspecific hybrid seeds in *V. vinifera*, to obtain larger seedling populations in a short time and enable more rapid grape breeding.

The cultivation of seeds into seedlings is one of the most important determinants of breeding efficiency. The germination rate of grape seeds is typically low, 30–50%, with a final seedling rate that is even lower [5,6,7]. Low germination rates can be due to the selection of parents, an incompletely developed seed embryo of the female parent, or a hard seed coat that is not easy to crack [8]. During seed collection and storage, seeds that are not fully mature or subjected to improper temperature and humidity during storage can result in seed decay or premature germination [9]. If the stratification time is too long or too short, the phenolic compounds in seeds can inhibit the germination rate [10]. Humidity can be difficult to control when trying to germinate seeds. If the humidity is too low, the germination rate will be low due to a lack of water, but if the humidity is too high, seeds are prone to mold and rot [5]. After sowing, the seedling stage may suffer blight or be inhibited by improper cultivation conditions [11]. Previous studies on grape seed germination and seedling formation examined differences of germination rates among different populations and varieties, differences of seedling formation rates among different cultivation and transplanting methods or characterized the physiology of germination and the optimal way to release dormancy [12,13]. Chai et al. compared the germination and seedling rates of cultivars *V. vinifera*, *V. labrusca*, and Franco-American, with *V. vinifera* and Franco-American varieties displaying higher and lower germination rates, respectively [2,5]. Grape seeds experience physiological dormancy, and the dormancy degree and method of dormancy release differ among different varieties [14,15,16]. Pan et al. studied the effects of gibberellin acid (GA_3_) on the seed germination characteristics of *V. adenoclada* Hand. -Mazz, *V. davidi*, wine grape, and table grape, finding that GA_3_ significantly improved the germination rate, germination potential, and germination index of grape seeds, and shortened the length of germination [9,17,18,19]. In addition, effects of 6-benzyl aminopurine (6-BA), Forchior fennron (CPUU), acetic acid (HAc), polyethylene glycol, indole acetic acid, 2,4-dichlorophenoxyacetic acid, lime nitrogen, ammonium nitrate, and other chemicals on seed germination rate were described [6,8,20,21,22]. Zhang et al. investigated the effects of direct sowing seeds in field or in a greenhouse and evaluated the use of film mulching of seedlings in the field and in a greenhouse. The highest formation rate of hybrid seedlings was obtained by sowing seeds in a greenhouse with the hole disc method and then transplanting the seedlings to the field in mid to late May after the growth of 4–5 true leaves [7].

An understanding of dormancy, germination, and seedling formation in grape seeds remains lacking, and more comprehensive studies are needed. In this study, six grape varieties were used as intraspecific hybrid parents. Single factor and orthogonal test systems were used to analyze the influences of different stratification methods of chilling gauze-storage and chilling sand-storage on germination and seedling rates, the effects of chemical treatments on seed dormancy before germination, the influence of cutting beak on germination and seedling rates, and the efficacy of pre-germination methods using petri dishes or a bean sprouter to improve germination and seedling rates. The aim of this study was to provide technical reference to improve the germination and seedling formation of the intraspecific hybrid progenies of six grape varieties of *V. vinifera* and increase the efficiency of recurrent selection.

## 2. Results

### 2.1. Effects of Single Factor Treatment on Seed Germination Characteristics

**Figure 1:**
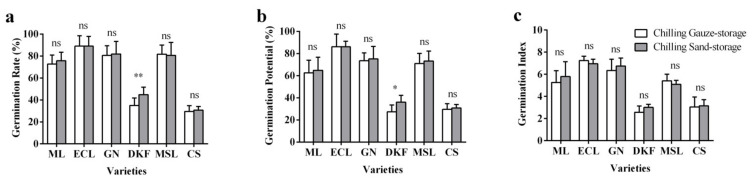
Effect of stratification treatments on seed germination characteristics of six tested varieties: (**a**) influence of stratification treatments on seed germination rate of tested varieties, (**b**) influence of stratification treatments on seed germination potential of tested varieties, and (**c**) influence of stratification treatments on seed germination index of tested varieties. Data in this figure were tested by Student’s t test; * *p* < 0.05 and ** *p* < 0.01 represent significant differences between treatments, and ns indicates not significant. ML, ECL, GN, DKF, MSL, and CS represent varieties of Meili, Ecolly, Garanior, Dunkelfelder, Marselan, and Cabernet Sauvignon.

As can be observed in Figure 1, compared with chilling gauze-storage, chilling sand-storage significantly improved the seed germination rate and potential of Dunkelfelder, but had no significant effect on the seed germination rate, potential, or index of Meili, Ecolly, Garanior, Marselan, and Cabernet Sauvignon.

**Figure 2:**
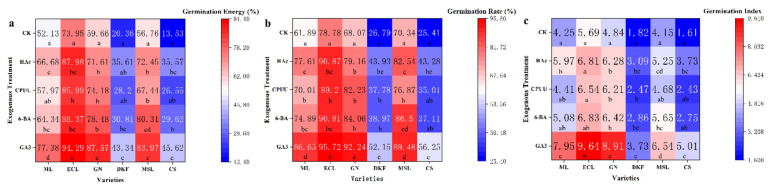
Effect of chemical treatments on seed germination characteristics of six tested varieties: (**a**) influence of chemical treatments on seed germination rate of tested varieties, (**b**) influence of chemical treatments on seed germination potential of tested varieties, and (**c**) influence of chemical treatments on seed germination index of tested varieties. Data in this figure were tested by One-way ANOVA; means followed by the same letter in a column do not differ according to Tuker’s test (*p* ≤ 0.05). ML, ECL, GN, DKF, MSL, and CS represent varieties of Meili, Ecolly, Garanior, Dunkelfelder, Marselan, and Cabernet Sauvignon.

It can be observed in Figure 2 that GA_3_, 6-BA, CPUU, and HAc improved seed germination rate, potential, and index of the six tested varieties. The effects of GA_3_ were the highest, especially for Ecolly, with seed germination rate, potential, and index of 95.72%, 94.29%, and 9.64% respectively.

**Figure 3:**
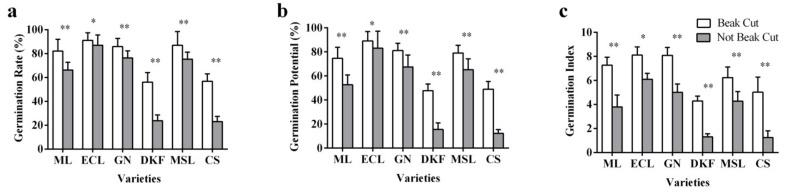
Effect of beak cutting treatment on seed germination characteristics of six tested varieties: (**a**) influence of beak cutting on seed germination rate of tested varieties, (**b**) influence of beak cutting on the germination potential of the tested varieties, and (**c**) influence of beak cutting on the germination index of the tested varieties. Data in this figure were tested by Student’s t test; * *p* < 0.05 and ** *p* < 0.01 represent significant differences between treatments, and ns indicates not significant. ML, ECL, GN, DKF, MSL, and CS represent varieties of Meili, Ecolly, Garanior, Dunkelfelder, Marselan, and Cabernet Sauvignon.

As displayed in Figure 3, beak cutting treatment improved seed germination rate, potential, and index of the six tested varieties, with very significant effects for Meili, Garanior, Dunkelfelder, Marselan, and Cabernet Sauvignon, and significant effects for Ecolly.

**Figure 4:**
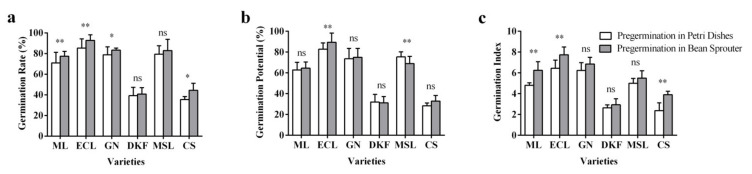
Effect of pre-germination treatments on seed germination characteristics of six tested varieties: (**a**) influence of pre-germination treatments on seed germination rate of tested varieties, (**b**) influence of seed germination treatments on seed germination potential of tested varieties, and (**c**) influence of pre-germination treatments on the germination index of tested varieties. Data in this figure were tested by Student’s t test; * *p* < 0.05 and ** *p* < 0.01 represent significant differences between treatments, and ns indicates not significant. ML, ECL, GN, DKF, MSL, and CS represent varieties of Meili, Ecolly, Garanior, Dunkelfelder, Marselan, and Cabernet Sauvignon.

The effects of pre-germination treatments on seed germination rate, potential and index varied for the six tested different varieties. As illustrated in Figure 4a, the two pre-germination methods had no significant effect on the seed germination rate of Dunkelfelderand and Marselan but resulted in extremely significant differences in the seed germination rates of Meili and Ecolly, and a significant difference in the seed germination rate of Garanior and Cabernet Sauvignon. As illustrated in Figure 4b, there was no difference in the effects of two pre-germination methods on the seed germination potential of Meili, Garanior, Dunkelfelder, and Cabernet Sauvignon, but very significant differences were found for Ecolly and Marselan. As displayed in Figure 4c, there was no difference for the two pre-germination methods on the seed germination index of Garanior, Dunkelfelderand, and Marselan, but a very significant difference for Meili, Ecolly, and Cabernet Sauvignon.

### 2.2. Effects of Single Factor Treatment on Seed Seedling Characteristics of 6 Tested Varieties

**Figure 5:**
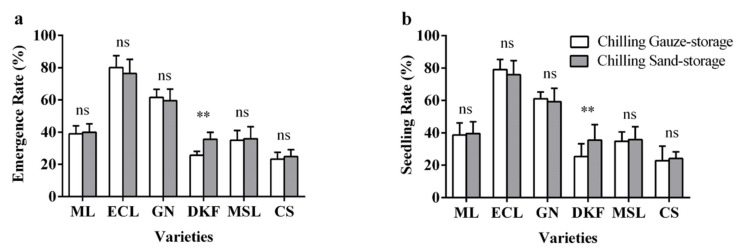
Effect of stratification treatments on seed seedling characteristics of six tested varieties: (**a**) influence of stratification treatments on seed seedling rate of tested varieties, and (**b**) influence of stratification treatments on seed seedling rate of tested varieties. Data in this figure were tested by Student’s t test; ** *p* < 0.01 represent significant differences between treatments, and ns indicates not significant. ML, ECL, GN, DKF, MSL, and CS represent varieties of Meili, Ecolly, Garanior, Dunkelfelder, Marselan, and Cabernet Sauvignon.

As illustrated in Figure 5, there was no significant difference in the effects of the two stratification treatments on the emergence rate and seedling rate of Meili, Ecolly, Garanior, Marselan, and Cabernet Sauvignon, but there were very significant effects for Dunkelfelder.

**Figure 6:**
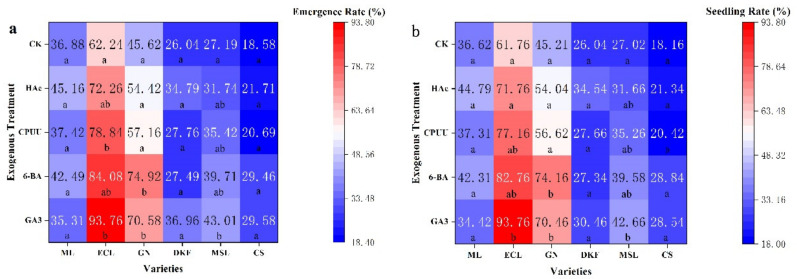
Effect of chemical treatments on seed seedling characteristics of six tested varieties: (**a**) effect of chemical treatments on seedling rate of tested varieties, and (**b**) effect of chemical treatments on seed seedling rate in tested varieties. Data in this figure were tested by One-way ANOVA; means followed by the same letter in column do not differ according to Tuker’s test (*p* ≤ 0.05). ML, ECL, GN, DKF, MSL, and CS represent varieties of Meili, Ecolly, Garanior, Dunkelfelder, Marselan, and Cabernet Sauvignon.

As demonstrated in Figure 6, different chemical treatments improved the seed emergence and seedling rates of Ecolly, Garanior, Dunkelfelder, Marselan, and Cabernet Sauvignon seeds. Figure 6a illustrates that there was no difference in the effects of different chemical treatments on the seed emergence rate of Meili, Dunkelfelder, and Cabernet Sauvignon. For all but Meili, the seed emergence rate was highest for the GA_3_ treated seeds, and for Ecolly, the seed emergence rate was highest, 93.76%. The same effects were observed for the seedling rate. As displayed in Figure 6b, there was no difference in the effects of different chemical treatments on the seed seedling rate for Meili, Dunkelfelder, and Cabernet Sauvignon. For all varieties but Meili, the seed seedling rate of GA_3_ treated seeds was the highest, especially for Ecolly, seed seedling rates reached 93.76%.

**Figure 7:**
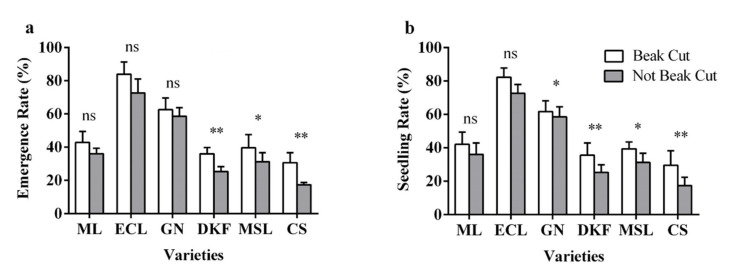
Effect of beak cutting treatment on seedling characteristics of six tested varieties: (**a**) influence of beak cutting treatment on emergence rate of tested varieties, and (**b**) influence of beak cutting treatment on seedling rate of tested varieties. Data in this figure were tested by Student’s t test; * *p* < 0.05 and ** *p* < 0.01 represent significant differences between treatments, and ns indicates not significant. ML, ECL, GN, DKF, MSL, and CS represent varieties of Meili, Ecolly, Garanior, Dunkelfelder, Marselan, and Cabernet Sauvignon.

It can be observed in Figure 7 that beak cutting treatment increased the seed emergence rate and seedling rate for the six tested varieties. As presented in Figure 7a, there was no difference in the effect of beak cutting treatment on the seed emergence rate of Meili, Ecolly, and Garanior, but a very significant effect was found for Dunkelfelder and Cabernet Sauvignon, and a significant effect was observed for Marselan. As displayed in Figure 7b, there was no difference in the effect of beak cutting treatment on the seedling rate of Meili and Ecolly, a significant effect for Garanior and Marselan, and a very significant effect for Dunkelfelder and Cabernet Sauvignon.

**Figure 8:**
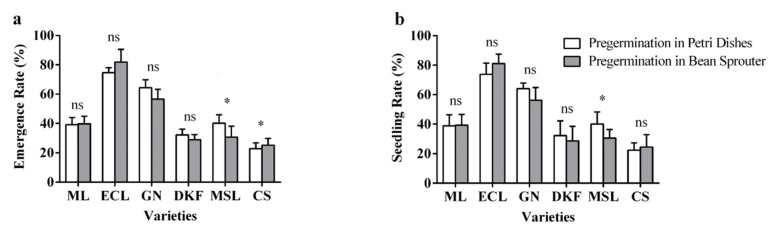
Effect of pre-germination treatments on seed seedling characteristics of tested varieties: (**a**) influence of pre-germination treatments on seed emergence rate of tested varieties, and (**b**) influence of pre-germination treatments on seed seedling rate of tested varieties. Data in this figure were tested by Student’s t test; * *p* < 0.05 represent significant differences between treatments, and ns indicates not significant. ML, ECL, GN, DKF, MSL, and CS represent varieties of Meili, Ecolly, Garanior, Dunkelfelder, Marselan, and Cabernet Sauvignon.

It can be observed in Figure 8 that pre-germination treatments had varied effects on seed emergence and seedling rates for the six tested varieties. As displayed in Figure 8a, there was no difference between the two pre-germination methods on the seed emergence rates of Meili, Ecolly, Garanior, and Dunkelfelder, but a significant difference for Marselan and Cabernet Sauvignon. As illustrated in Figure 8b, there was no difference between the two pre-germination methods on the seedling rate for Meili, Ecolly, Garanior, Dunkelfelder, and Cabernet Sauvignon, but a significant difference was found for Marselan.

### 2.3. Effects of Composite Treatments on Seed Germination and Seedling Characteristics

Using the seed germination and emergence rates as evaluation indexes, the orthogonal test was performed. The results for Meili seeds are presented in Table 1. Ki represents the average number of germination or emergence rates corresponding to any column with level number i (i = 1, 2, 3, 4, 5), and range R represents the difference between the maximum and minimum values of Ki in any column. The best treatment combination of seed germination and seedling formation of each variety can be obtained by range analysis.

For Meili seeds, factor A’s influence on the germination and emergence rates ranks fourth, but for the germination rate, A2 was taken and for the emergence rate, A1 was taken. For A1, the germination rate was reduced by 3.46% and the emergence rate was increased by 1.10%, so factor A is A2. For factor B, the influence on the germination and emergence rates ranked first, but for the germination rate, B1 was taken and for the emergence rate, B4 was taken. For B1, the germination rate was increased by 6.11% and the emergence rate was reduced by 19.92%, so factor B is B4. Factor C’s influence on the germination and emergence rates ranks second, and both take C1, so factor C takes C1. For factor D, the influence on the germination and emergence rates ranked third, and D2 was taken, so factor D was taken as D2. Therefore, for Meili seeds, this results in an optimal combination of A2B4C1D2, that is, chilling sand-storage + HAc soaking seed + beak cutting + pre-germination in petri dishes.

For Ecolly seeds (Table 2), factor A’s influence on the germination and emergence rates ranked third, and A2 was taken, so factor A is A2. For factor B, the influence on the germination and emergence rates ranked first, and both take B1, so factor B takes B1. Factor C’s influence on the germination and emergence rates ranked fourth, but for the germination rate, C1 was taken and for the emergence rate, C2 was taken. For C1, the germination rate was increased by 2.90% and the emergence rate was reduced by 2.39%, so factor C is C1. For factor D, the influence on the germination and emergence rates ranked second, but for the germination rate, D2 was taken and for the emergence rate, D1 was taken. For D1, the germination rate was reduced by 6.23% and the emergence rate was increased by 24.86%, so factor D is D1. Therefore, for Ecolly seeds, this results in an optimal combination of A2B1C1D1, that is, chilling sand-storage + GA_3_ soaking seed + beak cutting + pre-germination in a bean sprouter.

For Garanior seeds (Table 3), factor A’s influence on the germination and emergence rates ranks fourth and third respectively, and A2 was taken, so factor A is A2. For factor B, the influence on the germination and emergence rates ranks first, but for germination rate, B1 was taken and for emergence rate, B4 was taken. For B1, the germination rate was increased by 4.80% and the emergence rate was reduced by 6.15%; B4 was taken. Factor C’s influence on the germination and emergence rates ranks second, but for the germination rate, C1 was taken and for emergence rate, C2 was taken. For C1, the germination rate was increased by 12.62% and the emergence rate was reduced by 14.48%, so factor C is C2. Factor D’s influence on germination and emergence rates ranks third and fourth respectively, but for the germination rate, D2 was taken and for the emergence rate, D1 was taken. For D1, the germination rate was reduced by 6.22% and the emergence rate was increased by 3.19%, so factor D is D2. Therefore, for Garanior seeds, this results in an optimal combination of A2B4C2D2, that is, chilling sand-storage + HAc soaking seed + no beak cutting + pre-germination in petri dishes.

For Dunkelfelder seeds (Table 4), factor A’s influence on the germination and emergence rates ranks fourth and second respectively, and A2 was taken, so factor A is A2. For factor B, the influence on the germination and emergence rates ranks first, and both take B1, so factor B is B1. For factor C, the influence on the germination and emergence rates ranks second and fourth respectively, and both take C1, so factor C is C1. For factor D, the influence on the germination and emergence rates ranks third, but the germination rate, D2 was taken and for emergence rate, D1 was taken. For D1, the germination rate was reduced by 10.54% and the emergence rate was increased by 10.46%, so factor D is D2. Therefore, for Dunkelfelder seeds, this results in an optimal combination of A2B1C1D2, that is, chilling sand-storage + GA_3_ soaking seed + beak cutting + pre-germination in petri dishes.

For Marselan seeds (Table 5), factor A’s influence on the germination and emergence rates ranks second and fourth respectively, but for the influence on the germination rate, A1 was taken and for the emergence rate, A2 was taken. For A1, the germination rate was increased by 5.83% and the emergence rate was reduced by 2.50%, so factor A is A1. For factor B, the influence on the germination and emergence rates ranks first, but for the germination rate, B1 was taken and for the emergence rate, B2 was taken. For B1, the germination rate was increased by 4.97% and the emergence rate was reduced by 0.37%, so factor B is B1. Factor C’s influence on the germination and emergence rates ranks third, and both take C1, so factor C is C1. For factor D, its influence on the germination and emergence rates ranks fourth and second respectively, and both take D1, so factor D is D1; Therefore, for Marselan seeds, this results in an optimal combination of A1B1C1D1, that is, chilling gauze-storage + GA_3_ soaking seed + beak cutting + pre-germination in bean sprouter.

For Cabernet Sauvignon seeds (Table 6), factor A’s influence on the germination and emergence rates ranks fourth and third respectively, and A2 was taken, so factor A is A2. For factor B, the influence on the germination and emergence rates ranks second, but for the germination rate, B1 was taken and for emergence rate, B2 was taken. For B1, the germination rate was increased by 30.73% and the emergence rate was reduced by 25.32%, so factor B is B1. Factor C’s influence on the germination and emergence rates ranks first, and both take C1, so factor C is C1. For factor D, the influence on the germination and emergence rates ranks third and fourth respectively, and both take D2, so factor D is D2. Therefore, for Cabernet Sauvignon seeds, this results in an optimal combination of A2B1C1D2, that is, chilling sand-storage + GA_3_ soaking seed + beak cutting + pre-germination in petri dishes.

### 2.4. Verification Experiment

Since the optimal compound treatment predicted by the orthogonal experiment of Ecolly, Garanior, Dunkelfelder, and Cabernet Sauvignon grape seeds does not appear in the orthogonal experiment table, the germination and emergence rates of the seeds under this optimal treatment combination need to be verified, to ensure the authenticity of the experimental results. Therefore, it was verified by three sets of parallel experiments under the optimal compound treatment conditions, which are listed in Table 7. It can be observed in the table that the results of the verification experiment are representative, indicating that the optimal compound treatment is reasonable and feasible, and has good reproducibility.

## 3. Discussion

### 3.1. Effects of Exogenous Treatment on Seed Germination Characteristics

Temperature and humidity are key factors for seed germination and also important environmental conditions for seed germination effect [23,24]. Different treatments may be more effective for different varieties based on differences in morphological structure and growth characteristics [25]. The results demonstrated significant effects on the germinating rate and potential of Dunkelfelder by chilling sand-storage. The seed coat of Dunkelfelder is hard, and humidity is low during stratification. This could limit seed expansion, resulting in slow germination and low germination rate [26,27]. Chemical treatments can change the internal metabolic mechanism of seeds, relieve seed dormancy, and promote seed germination [28,29]. The results of this study demonstrated that GA_3_, 6-BA, CPUU, and HAc improved the germination rate, potential, and index of grape seeds of the tested varieties, with GA_3_ providing the largest treatment effect. This is consistent with the results of previous studies [8,9,20,21,30]. This may be because GA_3_, as a plant growth regulator, participates in the signal transmission during seed germination, and improves the activity of various enzymes to accelerate the decomposition and synthesis of internal substances in seeds, to improve the germination rate, potential, and index of the seeds [31,32,33]. Beak-cutting treatments also improved the germination rate, potential, and index of the tested varieties, which was consistent with the results of previous studies [21]. During seed germination, the control of humidity is particularly important. When the humidity is too low, the seed germination rate will be affected because of water limitation, but high humidity can make seeds become moldy and rotten [5]. Pre-germination in a bean sprouter also improved germination, with different effects observed for different varieties. This effect may be related to the constant humidity during pre-germination in a bean sprouter [30].

### 3.2. Effects of Exogenous Treatments on Seed Seedling Characteristics

Seed germination is the first stage of life in which plants can perceive the external environment, and this is also the most sensitive stage to environmental changes [34]. Germination directly affects seedling emergence and subsequent growth [35]. There were very significant differences in the influence of the two stratification methods on the emergence and seedling rates of Dunkelfelder. This may be due to the better relative swelling effect of seeds in the sand storage process, which improved germination and seedling formation [26]. Varieties with high germination efficiency and rate can more easily produce seedlings under appropriate environmental conditions [36]. There was no difference in the effects of different chemical treatments on the emergence and seedling rates of Meili, Dunkelfelder, and Cabernet Sauvignon, but there were different effects on the emergence and seedling rates of Ecolly, Garanior, and Marselan, potentially related to the relatively higher germination rate and potential of these varieties. Seed germination and seedling growth are the beginning stage of the plant life cycle, and exogenous treatment affects seed vigor [37,38]. Beak-cutting treatments improved the germination rate, potential, and index of the six tested varieties, but also improved the emergence and seedling rates, consistent with the results of previous studies [21,39]. Increasing the seedling rate can conserve resources [40]. The emergence and seedling rates of Ecolly and Cabernet Sauvignon were increased by pre-germination in a bean sprouter, but these rates were decreased for Garanior, Dunkelfelder, and Marselan. This may be related to differences between varieties, such as seed maturity, endogenous hormone content, seed coat thickness, thousand-grain weight, size, water content, dormancy type, and the amount of cold needed to break dormancy [7,15,16,41,42]. In addition, we found that for all exogenous treatments, the seedling and emergence rates of the six tested varieties had a similar ratio, which may be related to the use of a seed coating agent before germination. Previous studies have found that a seed coating agent exhibits a good prevention and control effect on seedling stage wilt disease, which greatly increases the seedling rate [8].

### 3.3. Effects of Compound Treatments on Seed Germination and Seedling Characteristics

Seed germination and seedling growth are key stages of plant growth and development but are also the most vulnerable. Germination rate, potential, and index, and emergence and seedling rate are important indicators of seed germination and seedling growth, which together determine the seedling efficiency [43]. In conventional hybrid breeding and seed selection, the phenotypic traits of seeds drive the selection of high-quality strong seedlings, and the germination and seedling characteristics of seeds allow the selection of the best female parent for hybrid breeding [2]. The results of this study demonstrated that the optimal combination of treatments for Dunkelfelder and Cabernet Sauvignon was chilling sand-storage + GA_3_ soaking seed + beak cutting + pre-germination in petri dishes. The optimal treatment combination for Meili was chilling sand-storage + HAc soaking seed + beak cutting + pre-germination in petri dishes. The optimal treatment combination for Ecolly was chilling sand-storage + GA_3_ soaking seed + beak cutting + pre-germination in a bean sprouter. The optimal treatment combination for Garanior was chilling sand-storage + HAc soaking seed + no beak cutting + pre-germination in petri dishes. The optimal treatment combination for Marselan was chilling gauze-storage + GA_3_ soaking seed + beak cutting + pre-germination in a bean sprouter. Overall, for Meili, Ecolly, Garanior, Dunkelfelder, and Cabernet Sauvignon seeds, chilling sand-storage was more effective than chilling gauze-storage, which may be related to the more constant humidity of sand storage [7]. For chemical treatments, GA_3_ had larger effects for Ecolly, Dunkelfelder, Marselan, and Cabernet Sauvignon, and HAc had greater effects on Meili and Garanior. As an efficient and broad-spectrum plant growth regulator, GA_3_ can increase the gibberellin content in seeds after soaking, regulate the proportion balance between inhibitor and promoter in advance, and enhance germination and seedling formation [33,44]. Soaking seeds with acetic acid can reduce the barrier of the seed coat, with enhanced permeability for improved seedling germination rates [45]. Beak-cutting is better than no-beak-cutting for the seeds of Melly, Ecolly, Dunkelfelder, Marselan, and Cabernet Sauvignon, and no-beak-cutting is better than beak-cutting for Garanior seeds. This may be related to the thickness of the seed coat. Beak-cutting is performed to release the binding of the hard peel to the embryo. If the seed coat of the seed is thin, beak-cutting may reduce the germination and emergence rates of the seed by damaging the embryo [21]. For Ecolly and Cabernet Sauvignon seeds, pre-germination in a bean sprouter is better than pre-germination in petri dishes, and for Meili, Garanior, Dunkelfelder, and Marselanseeds, pre-germination in petri dishes is better than in a bean sprouter. This may be related to the characteristics of seed germination. In our research, the germination potential of Ecolly and Marselan seeds is higher. This is helpful for the seeds to complete the germination process as soon as possible, effectively avoiding moldy and rotten seeds under the condition of prolonged moist germination and reducing the germination rate of the seeds. In addition, the characteristics of seed germination and seedling formation are also related to the growth cycle of the variety and the hardness of the seed coat. Early-maturing varieties have a short growth cycle, and the embryo development is not complete. The seed coat is hard, and the seed embryo is easily confined by the hard outer shell. These are not conducive to the germination of seeds [9]. 

The optimal combination treatments for the six grape varieties differed due to differences in the seeds of the different varieties. Dunkelfelder and Cabernet Sauvignon are red medium or late ripening wine grape varieties, suggesting that the conditions identified here would be suitable for the treatment of other red medium and late ripening varieties. The germination and seedling characteristics of hybrid seeds are very similar to the characteristics of the female parent [8], so the optimal treatment for a female parent can provide reference for the germination and seedling formation of seeds produced by intraspecific hybrid combination, using the six tested varieties as parents. Further, the results of this study reveal higher germination and seedling rates for Ecolly, Garanior, and Marselan, with better consistency at the seedling stage, suggesting the suitability of these varieties for use as the female parent of grape hybridization.

## 4. Materials and Methods

### 4.1. Materials

With the primary research, the seeds of six grape varieties of *V. vinifera* were used as experimental materials: Ecolly, Meili, Garanior, Cabernet Sauvignon, Marselan, and Dunkelfelder (Table 8). The seeds were collected from an experimental vineyard of the Northwest Agriculture and Forestry University (NWAFU) located in Yangling of Shanxi Province (lat. 34° N, long. 108° E), China. This area has a semiarid continental monsoon climate, and the soil type is bauxite. Self-rooted vines of *V. vinifera*. were planted in 2013. Vine rows were oriented west-east, with vines spaced in 1.0 × 2.5 m rows. The vines were cordon-trained and pruned to two buds per spur. All viticultural practices were performed according to local standards. The characteristics of seeds of the six varieties are presented in Table 1.

### 4.2. Treatment Methods of Seeds

#### 4.2.1. Collection and Storage

Grapes were collected randomly from different vines and different parts of clusters after reaching full maturity. One cluster was randomly selected for each vine, and six grapes (two each from the upper, middle, and lower parts) were sampled from each chosen cluster. The pulp and peel of the collected grapes were removed, and the mature seeds with full shapes were rinsed several times with water. The washed seeds were sterilized with 1% sodium hypochlorite for 15 min, and then rinsed four times with sterile water. After natural drying in the shade, the seeds were put into nylon bags, hung in a ventilated place for air drying, and then stored for later use [9,16,49].

#### 4.2.2. Stratification Treatment

Two stratification treatments were tested. In the first, the seeds were mixed with 3–4 times volumes of wet sand and then chilled in a refrigerator. The sand humidity was 60–80%, the refrigerator temperature was about 4 °C, and the chilling sand-storage is carried out for three months [50]. In the other stratification treatment, the seeds were wrapped with wet gauze, sub-packed in plastic bags with air holes, and then stored in a 4 °C refrigerator for three months [30]. The temperature and humidity were frequently checked during storage to prevent seed decay and premature germination.

#### 4.2.3. Chemical Treatments

Different chemicals were tested using previously determined amounts and 8 h of soaking time: 150 mg/L GA_3_, 100 mg/L 6-BA, 200 mg/L CPUU, HAc at 1:70, or pure water as a control [8,9,20,21,30].

#### 4.2.4. Beak-Cutting Treatments

Before pre-germination, the seeds were sterilized, soaked in 1% sodium hypochlorite for 15 min, and then fully stirred with 25 g/L Syngenta fludioxonil seed coating agent (slurry: seeds of 1:50) [15,18]. Then the beak-cutting and non-beak-cutting treatments were performed for the pre-germination test.

#### 4.2.5. Pre-Germination Treatment

Two kinds of pre-germination treatment were tested. In the traditional method, the treated seeds were placed in petri dishes containing double-layer filter paper in the upper and lower parts and then placed in a light incubator to accelerate germination (the temperature of the light incubator is 25 °C, the filter paper is timely moisturizing, 12 h of light and 12 h of darkness) [17]. In the other method, the sterilized seeds were uniformly sown in a bean sprouter, and distilled water was added to accelerate germination (the temperature of the bean sprouter is 25 °C, intermittent spray moisturizing, 12 h of light and 12 h of darkness) [30].

#### 4.2.6. Sowing and Planting

Germination was measured by evaluating seed cracking and white exposure or long root. The number of sprouting seeds per day was determined for each treatment of different varieties. Germinated seeds were sown in the greenhouse using burrow plate seedling cultivation [2,7]. After seed emergence, wheat bran mixed with insecticides was sprinkled around the burrow tray to prevent insect pests. Carbendazim solution (1 g/L) was applied one or two times to prevent blight. Emergence was determined as the time when the seedlings had grown two pieces of cotyledon, and then the seedlings were moved into a 10 cm gallon pot (The growing medium is nutrient soil). When the seedlings had four true leaves and one heart seedling, the seedlings were counted [7].

### 4.3. Experimental Design

The study utilized a single factor experiment and an orthogonal experiment. The two experiments were conducted simultaneously, with three technical replicates for each treatment setting.

#### 4.3.1. Single Factor Experiment

The experiment was carried out to investigate the key steps of seed germination and seedling formation. The experiment included two stratification methods (chilling gauze-storage and chilling sand-storage), five chemicals (GA_3_, 6-BA, CPUU, HAc, and CK), beak-cutting or no beak-cutting before germination, and two pre-germination methods (pre-germination in petri dishes and pre-germination in a bean sprouter). The germination and seedling status were measured after sowing. Three biological replicates were performed, and 100 seeds were taken from each replicate.

#### 4.3.2. Orthogonal Experiment

The stratification, chemical substance, beak cutting, and pre-germination treatments were used as the four research factors, and the orthogonal experiment of composite treatments was designed using SPSS, as listed in Table 9. For each treatment, 100 seeds were collected, with three repeats. The germination and seedling formation were measured after sowing. Analysis was performed to determine the optimal compound treatment conditions.

#### 4.3.3. Verification Experiment

The experiment was repeated three times under the optimal compound treatment conditions of the orthogonal experiment to verify the results.

### 4.4. Assay Method of Primary Indicators

The germination rate (%) equals the total number of germinated seeds divided by the total number of seeds tested × 100%. The germination potential (%) is the number of seeds germinated within eight days of germination divided by the total number of seeds tested × 100%. The germination index = Σ (Gt/Dt), where, Gt is the germination at different times, Dt is the number of germination days (in this study, the first eight days are calculated), and Σ is the sum. The emergence rate (%) equals the number of seedlings (two pieces of cotyledon) divided by the number of seeds sown × 100%. The seedling rate (%) equals the number of seedlings (number of four true leaves and one heart seedlings) divided by the number of seeds sown × 100%.

### 4.5. Data Processing

Microsoft Excel 2013 was used to record and process the original data and calculate the mean and range. An analysis of variance and multiple comparisons were performed in SPSS 22.0 statistical software. Data were averaged by treatment, and treatments (stratification treatment, beak-cutting treatments, and pre-germination treatments) were compared using the Student’s t test. The multiple comparisons tests (chemical treatments) were performed between treatments with Tukey’s test (* *p* < 0.05, ** *p* < 0.01). Origin 9.0 and GraphPad Prism 6 were used to present the results.

## 5. Conclusions

In this study, single factor and composite orthogonal experiments were used to determine the optimal pre-treatment methods for seed germination and emergence of six *V. vinifera* varieties. The single factor experiment demonstrated that two stratification methods (chilling gauze-storage and chilling sand-storage) and two pre-germination methods (pre-germination in petri dishes and pre-germination in a bean sprouter) vary in effectiveness for different varieties, so a simple and economical hierarchical or pre-germination method could be selected as needed. GA_3_ soaking and beak-cutting promote the germination and seedling rates of the tested varieties. The optimal way to improve the germination and emergence rates of the tested varieties was determined by a compound orthogonal experiment comparing several methods. The optimal treatment combination of Dunkelfelder and Cabernet Sauvignon were chilling sand-storage + GA_3_ soaking seed + beak cutting + pre-germination in petri dishes. The optimal treatment combination of Meili was chilling sand-storage + HAc soaking seed + beak cutting + pre-germination in petri dishes. The optimal treatment combination for Ecolly was chilling sand-storage + GA_3_ soaking seed + beak cutting + pre-germination in a bean sprouter. The optimal treatment combination of Garanior was chilling sand-storage + HAc soaking seed + no beak cutting + pre-germination in petri dishes. The optimal treatment combination for Marselan was chilling gauze-storage + GA_3_ soaking seed + beak cutting + pre-germination in a bean sprouter.

This study identified the optimal conditions for seed germination and seedling formation of six grape varieties, which will facilitate future work to characterize the seed germination and seedling formation of seeds, obtained by intraspecific hybridization of these varieties. This work also provides a reference for addressing problems of low seed germination rate and suboptimal seedling formation for better utilization of grape germplasms. However, this study only evaluated the effects of exogenous treatments on the germination, potential, index, emergence and seedling rates of the six tested varieties. The physiological changes inside seeds, the effect of seed embryo vigor, and the quality of seedlings after exogenous treatment were not studied in detail. Future work should determine the effects of physiological and biochemical reactions on seed germination and seedling formation characteristics of grapes.

## Figures and Tables

**Table 1:** Orthogonal experimental design and optimum results of Meili grape seeds.

Experiment Number		Experimental Factor				Experimental Result	
		A	B	C	D	Germination Rate (%)	Emergence Rate (%)
1		1	3	1	2	84.33	47.52
2		1	1	1	1	98.67	48.00
3		1	3	1	1	78.61	38.90
4		2	3	2	1	68.18	44.43
5		2	5	1	2	85.41	63.89
6		2	2	1	1	91.33	49.55
7		2	1	2	1	88.08	42.36
8		2	1	2	2	89.75	50.00
9		1	2	2	2	74.00	38.18
10		2	3	2	2	72.95	34.00
11		1	1	1	2	99.00	59.33
12		1	5	2	1	56.33	34.67
13		1	4	2	1	80.27	73.03
14		2	4	1	2	96.00	51.67
Germination rate (%)	k1	81.6	93.87	90.48	80.21		
	k2	84.53	82.67	75.65	85.92		
	k3		76.02				
	k4		88.13				
	k5		70.87				
	Range (R)	2.93	23	14.83	5.71		
	Primary and secondary order	B > C > D > A					
	Superior level	A2	B1	C1	D2		
	Excellent combination	A2B1C1D2					
Emergence rate (%)	k1	48.52	49.92	51.27	47.28		
	k2	47.99	43.86	45.24	49.23		
	k3		41.21				
	k4		62.35				
	k5		49.28				
	Range (R)	0.53	21.14	6.03	1.95		
	Primary and secondary order	B > C > D > A					
	Superior level	A1	B4	C1	D2		
	Excellent combination	A1B4C1D2					
Comprehensive optimum combination		A2B4C1D2					

A, B, C, and D represent experimental factor of stratification method, chemical substance, beat cutting, and pre-germination method respectively. 1, 2, 3, 4, and 5 correspond to the level factors of each experimental factor.

**Table 2:** Orthogonal experimental design and optimum results of Ecolly grape seeds.

Experiment Number		Experimental Factor				Experimental Result	
		A	B	C	D	Germination Rate (%)	Emergence Rate (%)
1		1	3	1	2	91.33	21.00
2		1	1	1	1	95.33	48.03
3		1	3	1	1	84.00	43.00
4		2	3	2	1	87.88	40.50
5		2	5	1	2	88.51	38.11
6		2	2	1	1	90.82	35.60
7		2	1	2	1	92.49	65.93
8		2	1	2	2	97.67	37.33
9		1	2	2	2	91.67	34.83
10		2	3	2	2	91.87	20.33
11		1	1	1	2	97.00	39.00
12		1	5	2	1	76.81	30.71
13		1	4	2	1	86.26	36.48
14		2	4	1	2	96.33	35.01
Germination rate (%)	k1	88.91	95.62	91.9	87.66		
	k2	92.22	91.25	89.24	93.48		
	k3		88.77				
	k4		91.3				
	k5		82.66				
	Range (R)	3.31	12.96	2.67	5.83		
	Primary and secondary order	B > D > A > C					
	Superior level	A2	B1	C1	D2		
	Excellent combination	A2B1C1D2					
Emergence rate (%)	k1	36.15	47.57	37.11	42.89		
	k2	38.97	35.22	38.02	32.23		
	k3		31.21				
	k4		35.75				
	k5		34.41				
	Range (R)	2.82	16.37	0.91	10.66		
	Primary and secondary order	B > D > A > C					
	Superior level	A2	B1	C2	D1		
	Excellent combination	A2B1C2D1					
Comprehensive optimum combination		A2B1C1D1					

A, B, C, and D represent experimental factor of stratification method, chemical substance, beat cutting, and pre-germination method respectively. 1, 2, 3, 4, and 5 correspond to the level factors of each experimental factor.

**Table 3:** Orthogonal experimental design and optimum results of Garanior grape seeds.

Experiment Number		Experimental Factor				Experimental Result	
		A	B	C	D	Germination Rate (%)	Emergence Rate (%)
1		1	3	1	2	85.24	66.74
2		1	1	1	1	96.49	48.20
3		1	3	1	1	84.71	51.33
4		2	3	2	1	83.08	59.77
5		2	5	1	2	87.92	54.27
6		2	2	1	1	90.77	51.59
7		2	1	2	1	86.33	72.00
8		2	1	2	2	91.19	72.00
9		1	2	2	2	81.00	52.15
10		2	3	2	2	78.69	51.69
11		1	1	1	2	97.62	49.33
12		1	5	2	1	55.46	41.37
13		1	4	2	1	81.68	81.78
14		2	4	1	2	95.22	46.90
Germination rate (%)	k1	83.17	92.91	91.14	82.65		
	k2	87.6	85.89	79.63	88.13		
	k3		82.93				
	k4		88.45				
	k5		71.69				
	Range (R)	4.43	21.22	11.51	5.48		
	Primary and secondary order	B > C > D > A					
	Superior level	A2	B1	C1	D2		
	Excellent combination	A2B1C1D2					
Emergence rate (%)	k1	55.84	60.38	52.62	58.01		
	k2	58.32	51.87	61.54	56.15		
	k3		57.38				
	k4		64.34				
	k5		47.82				
	Range (R)	2.47	16.52	8.91	1.85		
	Primary and secondary order	B > C > A > D					
	Superior level	A2	B4	C2	D1		
	Excellent combination	A2B4C2D1					
Comprehensive optimum combination		A2B4C2D2					

A, B, C, and D represent experimental factor of stratification method, chemical substance, beat cutting, and pre-germination method respectively. 1, 2, 3, 4, and 5 correspond to the level factors of each experimental factor.

**Table 4:** Orthogonal experimental design and optimum results of Dunkelfelder grape seeds.

Experiment Number		Experimental Factor				Experimental Result	
		A	B	C	D	Germination Rate (%)	Emergence Rate (%)
1		1	3	1	2	53.59	35.02
2		1	1	1	1	81.22	55.52
3		1	3	1	1	54.02	28.00
4		2	3	2	1	59.68	50.20
5		2	5	1	2	52.85	78.62
6		2	2	1	1	73.77	45.33
7		2	1	2	1	46.85	81.25
8		2	1	2	2	55.70	44.67
9		1	2	2	2	40.00	23.33
10		2	3	2	2	52.00	27.33
11		1	1	1	2	92.89	50.00
12		1	5	2	1	24.06	18.00
13		1	4	2	1	39.73	51.11
14		2	4	1	2	77.00	36.00
Germination rate (%)	k1	55.07	69.16	69.33	54.19		
	k2	59.69	56.88	45.43	60.58		
	k3		54.82				
	k4		58.37				
	k5		38.45				
	Range (R)	4.62	30.71	23.9	6.39		
	Primary and secondary order	B > C > D > A					
	Superior level	A2	B1	C1	D2		
	Excellent combination	A2B1C1D2					
Emergence rate (%)	k1	37.28	57.86	46.93	47.06		
	k2	51.91	34.33	42.27	42.14		
	k3		35.14				
	k4		43.56				
	k5		48.31				
	Range (R)	14.63	23.52	4.66	4.92		
	Primary and secondary order	B > A > D > C					
	Superior level	A2	B1	C1	D1		
	Excellent combination	A2B1C1D1					
Comprehensive optimum combination		A2B1C1D2					

A, B, C, and D represent experimental factor of stratification method, chemical substance, beat cutting, and pre-germination method respectively. 1, 2, 3, 4, and 5 correspond to the level factors of each experimental factor.

**Table 5:** Orthogonal experimental design and optimum results of Marselan grape seeds.

Experiment Number		Experimental Factor				Experimental Result	
		A	B	C	D	Germination Rate (%)	Emergence Rate (%)
1		1	3	1	2	91.41	48.50
2		1	1	1	1	98.67	72.00
3		1	3	1	1	90.61	48.00
4		2	3	2	1	84.17	56.02
5		2	5	1	2	66.49	22.00
6		2	2	1	1	94.00	79.43
7		2	1	2	1	94.33	49.50
8		2	1	2	2	98.00	72.00
9		1	2	2	2	91.47	43.44
10		2	3	2	2	69.14	33.33
11		1	1	1	2	99.33	51.33
12		1	5	2	1	74.39	48.70
13		1	4	2	1	84.09	45.62
14		2	4	1	2	87.09	54.48
Germination rate (%)	k1	90	97.58	89.66	88.61		
	k2	84.75	92.74	85.09	86.13		
	k3		83.83				
	k4		85.59				
	k5		70.44				
	Range (R)	5.25	27.14	4.57	2.47		
	Primary and secondary order	B > A > C > D					
	Superior level	A1	B1	C1	D1		
	Excellent combination	A1B1C1D1					
Emergence rate (%)	k1	51.09	61.21	53.68	57.04		
	k2	52.39	61.44	49.8	46.44		
	k3		46.46				
	k4		50.05				
	k5		35.35				
	Range (R)	1.31	26.09	3.88	10.59		
	Primary and secondary order	B > D > C > A					
	Superior level	A2	B2	C1	D1		
	Excellent combination	A1B1C1D1					
Comprehensive optimum combination		A1B1C1D1					

A, B, C, and D represent experimental factor of stratification method, chemical substance, beat cutting, and pre-germination method respectively. 1, 2, 3, 4, and 5 correspond to the level factors of each experimental factor.

**Table 6:** Orthogonal experimental design and optimum results of Cabernet Sauvignon grape seeds.

Experiment Number		Experimental Factor				Experimental Result	
		A	B	C	D	Germination Rate (%)	Emergence Rate (%)
1		1	3	1	2	89.67	56.56
2		1	1	1	1	97.33	54.33
3		1	3	1	1	91.35	40.67
4		2	3	2	1	41.18	28.60
5		2	5	1	2	81.18	47.55
6		2	2	1	1	85.28	86.44
7		2	1	2	1	66.01	32.00
8		2	1	2	2	72.67	34.00
9		1	2	2	2	30.41	28.89
10		2	3	2	2	48.89	32.06
11		1	1	1	2	98.00	51.93
12		1	5	2	1	9.63	13.33
13		1	4	2	1	38.17	22.67
14		2	4	1	2	93.11	46.00
Germination rate (%)	k1	64.94	83.5	90.85	61.28		
	k2	69.76	57.84	43.85	73.42		
	k3		67.77				
	k4		65.64				
	k5		45.41				
	Range (R)	4.82	38.09	47	12.14		
	Primary and secondary order	C > B > D > A					
	Superior level	A2	B1	C1	D2		
	Excellent combination	A2B1C1D2					
Emergence rate (%)	k1	38.34	43.06	54.78	39.72		
	k2	43.81	57.67	27.36	42.43		
	k3		39.47				
	k4		34.33				
	k5		30.44				
	Range (R)	5.47	27.23	27.42	2.71		
	Primary and secondary order	C > B > A > D					
	Superior level	A2	B2	C1	D2		
	Excellent combination	A2B2C1D2					
Comprehensive optimum combination		A2B1C1D2					

A, B, C, and D represent experimental factor of stratification method, chemical substance, beat cutting, and pre-germination method respectively. 1, 2, 3, 4, and 5 correspond to the level factors of each experimental factor.

**Table 7:** Results of repeated experiment.

Varieties	Optimal Combination	Germination Rate	Emergence Rate
Ecolly	A2B1C1D1	94.21 ± 4.33	53.24 ± 4.19
Garanior	A2B4C2D2	92.18 ± 4.56	67.65 ± 5.34
Dunkelfelder	A2B1C1D2	79.99 ± 5.72	51.52 ± 4.24
Cabernet Sauvignon	A2B1C1D2	82.49 ± 7.01	56.17 ± 3.55

The data based on three replicates was represented as M ± SD. A, B, C, and D represent experimental factor of stratification method, chemical substance, beat cutting, and pre-germination method respectively. 1, 2, 3, 4, and 5 correspond to the level factors of each experimental factor.

**Table 8:** Characteristics of the six test *V. vinifera* varieties.

Variety	Breeding Method	Seed-Parent	Variety Type	Seed Characteristics
Meili [46]	Intraspecific current selection	[Muscat Hamburg × (Merlot × Riesling)] × (Muscat Hamburg, Merlot, Riesling)	Red mid-variety	Large seed, seed coat is medium thickness.
Ecolly [47]	Intraspecific current selection	[Chenin Blanc × (Chardonnay × Riesling)] × (Chenin Blanc, Chardonnay, Riesling)	White mid-variety	Small seed, seed coat is relatively thin.
Garanior [48]	Intraspecific hybridization	Gamay × Reichensteiner	Red early-maturing variety	Small seed, seed coat is relatively thin.
Dunkelfelder [47]	Intraspecific hybridization	Madeleine Angevine × Teinturier	Red mid-variety	Medium-sized seed, seed coat is relatively thin.
Marselan [47]	Intraspecific hybridization	Cabernet Sauvignon × Grenache	Red mid-late maturity variety	Small seed, seed coat is relatively thin.
Cabernet Sauvignon [47]	Intraspecific hybridization	Cabernet Franc × Sauvignon Blanc	Red late-maturing variety	Medium-sized seed, seed coat is medium thickness.

Meili and Ecolly selected the mixed pollen of the three parents in the process of intraspecific current selection. All tested varieties were obtained through intraspecific hybridization in *V. vinifera*.

**Table 9:** Level factors.

Level	Experimental Factor			
	Stratification Method (A)	Chemical Substance (B)	Beak Cutting (C)	Pre-Germination Method (D)
1	chilling gauze-storage	GA_3_	beak cutting	pre-germination in bean sprouter
2	chilling sand-storage	6-BA	no beak cutting	pre-germination in petri dishes
3		CPUU		
4		HAc		
5		CK		

## Data Availability

Data is contained within the article.
